# Noise Exposure and Hearing Capabilities of Quarry Workers in Ghana: A Cross-Sectional Study

**DOI:** 10.1155/2016/7054276

**Published:** 2016-01-21

**Authors:** Charles Kwame R. Gyamfi, Isaac Amankwaa, Frank Owusu Sekyere, Daniel Boateng

**Affiliations:** ^1^Center for Disability and Rehabilitation Studies, Department of Community Health, Kwame Nkrumah University of Science and Technology, Kumasi, Ghana; ^2^Department of Nursing, Garden City University College, Kumasi, Ghana; ^3^Department of Interdisciplinary Studies, University of Education, Kumasi, Ghana; ^4^Department of Community Health, Kwame Nkrumah University of Science and Technology, Kumasi, Ghana

## Abstract

*Introduction.* Although quarry operations have high economic significance, the effects they cause to the workers in terms of excessive noise production cannot be overlooked. This cross-sectional study assessed the extent of noise exposure and its influence on hearing capabilities among quarry workers in Ashanti region.* Methods*. The study involved 400 workers randomly selected from five quarries in Ashanti region from April to June 2012. Data was collected using structured questionnaires, physical examination, and audiological assessments. A logistic regression model was fitted to assess independent predictors of hearing loss.* Results*. All the machines used at the various quarries produced noise that exceeded the minimum threshold with levels ranging from 85.5 dBA to 102.7 dBA. 176 (44%) of study respondents had hearing threshold higher than 25 dBA. 18% and 2% of these were moderately (41–55 dBA) and severely (71–90 dBA) impaired, respectively. Age, duration of work, and use of earplugs independently predicted the development of hearing loss. Use of earplugs showed a protective effect on the development of hearing loss (OR = 0.45; 95% CI = 0.25, 0.84).* Conclusion. *This study provides empirical evidence on the extent of damage caused to quarry workers as a result of excessive noise exposure. This will support the institution of appropriate protective measures to minimize this threat.

## 1. Background

Occupational health is an important public health concern for the working population [1]. Various aspects of the working environment could expose an individual to potential hazardous elements. Noise is considered as one of these potential hazards and it is currently seen as a global health concern. Noise, which is defined loosely as annoying sounds, is part of the everyday human activity. Excessive noise beyond tolerated levels from all these sources is hazardous and could cause hearing impairment. This is a widespread occupational hazard, which could result in noise-induced hearing loss (NIHL). Other associated health effects include elevated blood pressure, sleeping difficulty, annoyance and stress, and temporary threshold shift (TTS) [2, 3].

The growing attention for NIHL is due to the fact that, unlike many injuries or illnesses, hearing loss may be permanent and irreversible. Exposure to excess noise is first observed as an increase in the threshold of hearing (threshold shift), as assessed by audiometry [3]. This is defined as a change in hearing thresholds of an average 10 dB or more at 2000, 3000, and 4000 Hz in either ear (poorer hearing) [4]. There are two types of NIHL, a temporary threshold shift, which is a temporary loss of hearing, and a permanent threshold shift, which involves a shift in the person's ability to hear soft sounds. This is as a result of long-term exposure to loud sounds of slightly lower intensity, such as factory noise or rock music.

Although noise is associated with almost every work activity, some activities are associated with particularly high levels of noise. In general, sounds above 85 dB are considered harmful, depending on how long and how often one is exposed to them and whether you wear hearing. Previous literature shows that workers in mines, quarries, sawmills, textile factors, printing presses, and many others work with machines that produces noise much higher than the tolerated levels and therefore expose workers to potential hearing loss [5–7]. In the large coal industry, about 76% are exposed to hazardous noise [8]. This causes about 25% of severe hearing problems and about 80% of hearing impairment in the workers' retirement age [8].

Although the situation could be improving in developed countries as a result of more widespread appreciation of the hazard and the institution of protective measures, evidence from developing countries suggests that average noise levels are well above the occupational level recommended in many developed nations [9, 10]. Increasing industrialization might exacerbate this situation in developing countries and therefore the need to assess the industrial noise pollution and its impact on the hearing capabilities of workers in such areas.

In Ghana, there is very little data on the effect of noise exposure on quarry industrial workers. More so, the increasing number of patients seeking medical attention for their ears at Hearing Assessment Centre at the Komfo Anokye Teaching Hospital (KATH) in Kumasi between 1995 to 1998 [11] has called for the need to focus attention on noise pollution on workers in the industrial settings such as quarry industries. Furthermore, no study has focused specifically on occupational hearing loss among workers of quarry industries even though the impact of noise pollution on hearing in mining company has been studied [11]. This study seeks to provide empirical evidence on the extent of noise exposure and its influence on hearing capabilities of workers in the quarry industry. This would help in formulating policies and institute preventive measures that will help minimize the risk of occupational hearing loss among the exposed population.

## 2. Methodology

### 2.1. Study Design and Settings

This was a descriptive cross-sectional study conducted in Kwabre East District of Ashanti region of Ghana. Kwabre District, carved out of the former Kwabre Sekyere District in 1988, is located almost in the central portion of Ashanti region. It is within latitude 6°44′′ north and longitude 1°33′′ to 1°44′′ west. The district shares common boundaries with Afigya-Sekyere District to the north, Kumasi metropolitan area to the south, Ejisu-Juaben District to the southeast, Atwima District to the west, and Offinso District to the northwest. The District has a total land area of 246.8 square kilometers constituting about 1.01% of the total land area of Ashanti region. Kwabre District is part of the Greater Kumasi City region, which is made up of Kumasi Metropolitan Area and the surrounding Districts. According to 2000's population and housing census, the district has a total population of about 205,372 people. The physiography of the area consists of scarps formed by the extensive quarry activities going on in the area. The main land usage in the area can be categorized into stone quarrying and agriculture. The quarries are mainly located near the mountains, where granite deposits have been detected. The quarry developers lease the land from the owners at a fee until the time the quarry work is finished. The main commercial purposes of quarrying are building stones and quarry dust. Agricultural activities in the area are mostly subsistence and cash crop farming.

### 2.2. Study Population and Sampling

The study population was drawn from workers of the five (5) quarry companies, namely, Northern mine quarry, A. Kannin mine, Taysec quarry company, KAS quarry company, and Siemens quarry company. Workers who have been working at the respective quarry for more than six months were included in the study. Workers who had previous history of hearing loss and those not directly involved in the quarrying process were excluded. The study comprised 400 workers from the various quarry companies. The composition of respondents from the quarry companies was as follows: A. Kannin (120 respondents; 20.1%), KAS quarry (80 respondents; 13.4%), Siemens quarry (60 respondents; 10%), Taysec (40 respondents; 6.7%), and Northern mines and quarry (98 respondents; 16.4%).

The surveys were conducted from April to June 2012. Based on the average number of clients per day for a specific facility and the desired sample size, a recruitment interval, *k*, was developed for each quarry. Based on this interval, the *k*th worker was interviewed on each interview day until the required sample size was reached for each quarry. The administration of the study was focused mainly on the industrial noise pollution and its impact on the hearing capabilities of workers. The desired sample size was calculated based on Kirkwood and Sterne [12], assuming a 40% proportion for the event of interest and 10% nonresponse rate.

### 2.3. Data Collection Techniques and Instruments

Data were collected mainly through interviewing. Participants were given consent form to sign and had all their concerns and questions answered before data collection began. All tools employed in the research were developed using standard procedures, pretested, and revised to ensure their validity and reliability and research assistants were trained to ensure uniformity in the administration of questionnaires. The various instruments used for measuring sound levels were the Casella sound level meter (in the recording of the noise levels in the various companies) and pure-tone audiometer (to evaluate the hearing threshold of subjects).

#### 2.3.1. Casella Sound Level Meter

The weighted networks in a meter are electronic circuits whose response to low frequencies and to very high frequencies is reduced in a specified manner. In general, four different weightings have been standardized internationally as A, B, C, and D. Network A is used in industrial setting and this was used throughout the study. To check for vibration, shock, and excessive heat, a calibrator was attached to the microphone of the meter and the reading was then compared with the calibrator's value. The meter was adjusted when required to bring it into calibration.

#### 2.3.2. Pure-Tone Audiometry

A biologic calibration of the pure-tone audiometer was performed daily using supposedly normal people with normal hearing prior to any audiological evaluation of the subjects. An otoscopic examination was also performed on each subject to exclude wax or any discharge in the ear canal or perforation of the eardrum to rule out possibility of conductive hearing loss. To overcome bias on the hearing acuity by ambient noise influence, five (5) of the subjects each from the various companies were randomly selected to undergo audiometric retesting at Hearing Assessment Centre at Komfo Anokye Teaching Hospital (KATH). The hearing thresholds obtained for these groups were found not to be different from those obtained within the premises of the factories. Audiometric tests were performed on employees working in the area with noise level exceeding the threshold limit value of 85 dBA using screening Audiometer, (AS608) obtained from the Hearing Assessment Centre of the Department of Ear, Nose and Throat (ENT) of KATH. Hearing acuity was measured at 5 dBA interval over a range of octave band frequencies from 500 to 8000 Hz. Hearing was considered normal if the threshold level was less than or equal to 25 dBA at the frequencies 250, 500, 1000, 2000, 4000, and 8000 Hz. However, the intensity of the stimuli was increased beyond 25 dBA at any frequency until a response was obtained. Individuals with a characteristic notch of four (4) KHz depicting the classical sign of NIHL were analysed. The degree and type of hearing loss were also determined using Goodman's [13] and Carhart's [14] approaches, respectively. Both ears of each subject were tested to establish pure-tone hearing sensitivity. All subjects were tested at the beginning of each work shift to ensure that those whose hearing had been “fatigued” might have gained some recovery after being away from the noise exposure.

### 2.4. Description of Variables and Data Analysis

The outcome variable was hearing threshold (dBA). Hearing threshold beyond 25 dBA is classified as having hearing loss and hearing loss was further classified based on its severity (26–40 dBA, mild; 41–55 dBA, moderate; 56–70 dBA, moderate-to-severe; 71–90 dBA, severe; and above 90, profound). The explanatory variables were age, duration of exposure, gender, and use of earplugs. The response of each subject and the data obtained from the administration of the physical instruments were scrutinized carefully and categorized in tables and graphs. Data was analyzed with R 3.1.1 [15]. The analysis involved a description of the baseline characteristics of respondents and the noise levels measured on various machines at the quarries. A chi-square test was conducted to see the association between workplace and hearing loss. Logistic regression analysis was done to look at the influence of age, duration of exposure, and use of earplugs on the odds of hearing loss among the quarry workers. Significance of associations was at *p* value of <0.05.

#### 2.4.1. Model Estimation and Assumptions

A correlation between age and duration of exposure was assumed and tested using Pearson's correlation. This was because the variable appeared to be approximately normally distributed. The explanatory variables included in the model were also tested for collinearity using the variance inflation factor (VIF). The choice of best-fitted model was based on Akaike's Information Criterion (AIC). This was proposed by Akaike [16] as a measure of the goodness of fit of an estimated statistical model. It takes into account both the statistical goodness of fit and the number of parameters that have to be estimated to achieve this particular degree of fit by imposing a penalty for increasing the number of parameters. The AIC is given as −2*∗*(log⁡likelihood) + 2*∗*(number  of  parameters  in  the  model), that is, −2*L* + 2*p*. Lower AIC is an indication of a better likelihood.

### 2.5. Ethical Consideration

A written permission was sought from the management of the various quarry companies and ethical clearance was granted by the Committee for Human Research and Population Ethics (CHRPE) at the School of Medical Science (SMS) of the Kwame Nkrumah University of Science and Technology (KNUST) and Komfo Anokye Teaching Hospital, Kumasi.

## 3. Results

### 3.1. Background Characteristics of Respondents

Majority of the respondents were males (81.4%). The mean age (SD) of the respondents was 41.7 (9.20). Most of the respondents were Christians (59%) and about 29% were Muslims. With respect to their level of education, 47.2% had junior secondary or middle school education. About 17% had tertiary education and 14 (2.3%) had no formal education. Majority had worked in the quarry for up to 10 years and 24.7% had worked in the quarry for less than 5 years. 132 (33%) of the respondents used earplugs and 61% of respondents who wore no earplugs had hearing thresholds above 25 dB as against 36% among those who wore earplugs.

### 3.2. Noise Measurement at Various Facilities under Study

All the companies had different production units with more or less the same type of machinery.  Table 1 displays the noise levels obtained from the machines in the various companies. The measurement values range from 85.5 dBA to 102.7 dBA, demonstrating that the noise levels produced exceed the limiting threshold level of 85 dBA. It was realized that all the five companies visited produced an excessive amount of noise capable of damaging the hearing status of the workers.

### 3.3. Hearing Thresholds at Various Quarries

**Figure 1:**
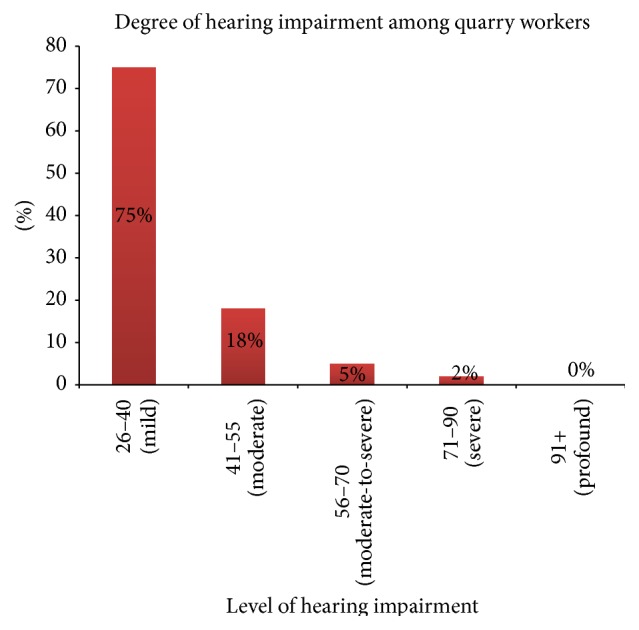
Level of hearing impairment among quarry workers.

The mean hearing threshold among all workers was 27.32 dBA and 176 respondents (44%) had hearing thresholds higher than 25 dBA. Comparatively, a higher mean threshold was observed among workers at Taysec company (29.98 dBA) followed by Northern mines and quarry (26.43 dBA) (Table 2). More than 50% of respondents from Taysec company and KAS company had hearing threshold of more than 25 dBA (58% and 69%, resp.). The proportion of respondents with hearing threshold > 25 dBA was comparatively low among A. Kannin and Siemens (29% and 33%, resp.). This difference in hearing thresholds among respondents from various working environments was statistically significant (*p* < 0.001), indicating the influence of the working environment on the hearing threshold of a respondent. As shown in Figure 1, majority of the respondents had mild hearing loss (132 out of 176 respondents). Thirty-two respondents (18%) had moderate hearing loss, whereas 4 (2%) respondents had severe cases of hearing loss.

### 3.4. Influence of Age, Duration of Exposure, and Use of Earplugs on Hearing Capabilities of Quarry Workers

**Figure 2:**
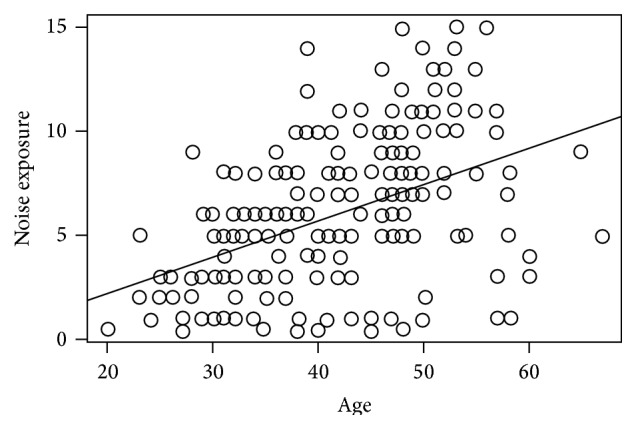
Scatterplot of age versus duration of exposure.

Table 3 presents results of the univariable and multivariable logistic regression analysis of factors influencing hearing loss among quarry workers. Two models were fitted in the multivariable analysis. In model 1, the place of work was adjusted for, while model 2 involved adjusting for place of work as well as adjusting for family history of hearing loss. The scatterplot of age and duration exposure showed a positive correlation, which was slightly moderate (*r* = 0.465) (Figure 2). The VIF for all variables (shown under Table 3) were very low and implied little or no existence of multicollinearity. To this end, all variables and an interaction between age and duration of exposure were included in the multivariable analysis. Based on the AIC, the best-fitted model (i.e., the model with the lowest AIC) in the multivariable analysis was a model without gender.

Age, duration of exposure, and use of earplugs significantly predicted hearing impairment among quarry workers (Table 3). In the univariable analysis, an increase in the age of quarry workers by one year resulted in 8% increase in the odds of hearing impairment (OR = 1.08; 95% CI = 1.05, 1.11). The odds of hearing impairment with respect to age increased to 1.15 in the multivariable analysis, where the place of work was adjusted for, and it remained unchanged in model 2. In model 1, an increase in duration of working at the quarry by one year results in about two times increase in the odds of hearing impairment (OR = 1.96; 95% CI = 1.2, 3.23). The strength of association, however, increased in model 2, indicating a possible confounding effect of the variables adjusted for in the association between duration of exposure and hearing impairment. The use of earplugs showed a protective effect on the odds of having hearing loss. In model 1, use of earplugs resulted in 54% decrease in odds of having NIHL (OR = 0.46; 95% CI = 0.26, 0.80) and this did not change much in model 2 (OR = 0.40). The interaction between age and duration of exposure was just significant with *p* value of 0.05.

## 4. Discussion

Occupational health is an important concern of the working person. Various elements concerning a person's working environment can predispose one to developing a disease process. Quarries are such organizations with high noise production levels as a result of their activities. The aim of this study was to look at NIHL among quarry workers in Ashanti region of Ghana. This study found a high prevalence of hearing impairment among the quarry workers and all machines used at the various sites produced noise levels that exceed the limiting threshold.

### 4.1. Noise Measurement at Various Facilities under Study

Results from this study indicate that all companies studied have different production units with similar type of machinery and the noise levels ranged from 85.5 dBA to 102.7 dBA. This reveals that all the machines used at the various companies produced sound that exceeds the minimum threshold of 85 dBA as recommended by World Health Organization [17], thereby having the potential of damaging the hearing status of workers. These noise levels also exceed the limit set by the Ghana Environmental Protection Agency (EPA) under the EPA Act of 1994 (Act 490) [18] which permits light industrial areas 70 dB during the day and 60 dB at night and heavy industrial areas 70 dB noise during the day and 70 dB noise at night. Consistent with this study, other studies from different countries reported exposure to high levels of noise pollution at the workplace. This includes the study by Ismail et al. [5] among quarry workers in Malaysia, where sound levels exceeded the level that may cause NIHL to the workers.

These, however, might not be limited to quarries alone. Studies on noise levels from other work settings including the study in Ghana by Boateng and Amedofu [6] on the impact of noise levels on hearing capabilities of workers in saw mills, corn mills, and printing houses revealed that noise level in corn mills exceeds the limiting value. Similarly, a study of working industries in Ethiopia by Mulugate [7] also reported noise levels higher than permissible levels of 90 dBA. Other studies from industrialized countries also indicate an overexposure to high noise levels among coalminers [8, 19]. These noise levels are, however, potentially hazardous and might result in hearing impairment among workers in that environment.

### 4.2. Noise Exposure and Hearing Loss

This study revealed that 44% of the quarry workers had hearing thresholds of more than 25 dB. This was, however, higher than the other prevalence reported from Sudan [20], 30.6%, and from studies by Amedofu [11], 23.0%, and Boateng and Amedofu [6], 23.0%. On the other hand, higher prevalence was observed by Chaddha and Singh (50.0%) [21] and Hong (60.0%) [22]. Three out of the five companies involved in the study also recorded a mean hearing threshold level of more than 25 dBA. All the companies had more than 25% of their respondents having NIHL with the percentage being highest among workers at KAS quarry. There was a significant association between the various working environments and HTL and this could be due to the type and quantities of noise generating equipment used in the various quarries and how these influence hearing capabilities. Analysis of the extent of hearing loss among respondents with HTL > 25 dB indicated that 75%, 18%, 5%, and 2% had mild, moderate, moderately severe, and severe hearing loss, respectively. These indicate the extent of ear damage caused to workers in severely noise-exposed environments like the quarry and the need to institute appropriate interventions to curb this.

### 4.3. Relationship between Age, Duration of Exposure, and Use of Earplugs and Hearing Loss among Respondents

Generally, there is an established association between the age of workers and hearing loss [23–25]. In this study, increasing age of quarry workers was associated with increasing odds of hearing loss among the quarry workers. This is consistent with the findings of an industry-specific study in USA [25], which showed that 90% of coal miners have hearing impairment by the age of 52 years. Also, it is estimated that 70% of male metal/nonmetal miners will have hearing impairment by the age of 60 years [26]. In the study by Ahmed et al. [27], age was the secondary predictor of hearing loss among workers in two plants in eastern Saudi Arabia.

The study also revealed a significantly positive association between duration of work at the quarry and hearing impairment. According to the United States National Institute of Deafness and Other Communicable Disorders [28], long or repeated exposure to sounds at or above 85 decibels can cause hearing loss. The louder the sound is, the shorter the time period before NIHL can occur becomes. Duration of exposure to noise at the working environment showed strong positive relationship with the hearing threshold among respondents in the multivariable analysis.

Wearing earplugs or other protective devices has been recommended for individuals involved in a loud activity. The proportion of respondents with hearing impairment was higher among those who did not wear earplugs. The multivariable analysis showed that use of ear protection decreased the odds of having hearing impairment among quarry workers. This is consistent with the outcome of the study by Hong [22], which found an inverse relationship between hearing protector use and hearing status of the employee. Ahmed et al. [27] also showed that wearing hearing protection devices is among the important factors that influence the measured hearing threshold values at low frequencies. Subjects who did wear hearing protection devices had lower measured hearing threshold values than subjects who did not wear hearing protection. According to a WHO bulletin on environmental burden of disease [3], the first priority in minimizing hearing loss is to reduce noise through technical measures such as introducing hearing protection for workers when engineering controls are not applicable or are insufficient. It was, however, advocated that the protective equipment must be properly selected, worn, and maintained.

## 5. Limitations

Although the study accessed the exposure to noise, there might still be other underlying factors, which might have contributed to hearing loss. These sources of noise produce what is called sociocusis and their effect on hearing loss might not differ from occupational hearing loss [1]. The authors believed that the inclusion of variables on other sources of loud noise measured major external sources of noise that could contribute to noise induced hearing loss. There was also a possibility of recall bias with respect to previous history of hearing problems. Research assistants were however trained to ensure comparability in the administration of questionnaires across the study centers.

## 6. Conclusion

This study found that most of the machines used in the quarry exceed the tolerable threshold of sound thereby having the potential of damaging the hearing status of workers. Noise levels measured among the quarries workers studied also exceed the limit set by the Ghana Environmental Protection Agency (EPA) under the EPA Act of 1994 (Act 490) which permits light industrial areas. It is recommended that EPA embark on regular monitoring to access noise levels and ensure that companies do not emit noise greater than the tolerable limits. Use of earplugs showed a protective effect on development of hearing loss. Efforts to ensure access and use of earplugs by quarry workers and quarry companies could be beneficial in reducing the absolute prevalence of hearing impairment especially among the elderly and long serving workers who have been shown to be at an increased risk of developing hearing loss.

## Figures and Tables

**Table 1:** Measured noise levels (in dBA) on selected machines in five quarry companies.

Type of machines	Quarry companies				
	A. Kannin	Kas	Taysec	Northern quarry	Siemens
	dBA	dBA	dBA	dBA	dBA
Drilling machine	89.4	96.2	95.0	97.0	94.9
Tyre wrench	87.0	86.3	87.9		88.5
Lathe machine	87.4		86.5		
Block making machine	100.3				
Generator set	86.5	97.9	87.9	94.3	
Crusher machine	99.6	102.5	101.3	100.4	102.7
Primary processing machine	89.9	91.0	98.0	96.8	98.8
Secondary processing machine	88.6	86.7	95.3	88.5	92.0
Excavator machine	97.1	95.6	94.9	89.5	88.6
Air compressor	87.4	86.9	93.2		95.4

**Table 2:** Hearing threshold levels among various groups of respondents.

Category of workers	Test results at 4 kz			Chi square/*p* value
	Mean HTL	HTL ≤ 25 dBA	HTL > 25 dBA	
		*N* (%)	*N* (%)	
Quarry workers				
KAS	33.91	25 (31)	55 (69)	36.37
Siemens	24.22	40 (67)	20 (33)	
A. Kannin	24.33	85 (71)	35 (29)	*p* < 0.001
Taysec	29.98	17 (43)	23 (58)	
Northern	26.43	57 (57)	43 (43)	

Total	27.32	224 (56)	176 (44)	

HTL, hearing threshold level.

**Table 3:** Logistic regression analysis of predictors of hearing loss (HTL > 25 dB) among quarry workers.

	Univariable		Multivariable	
Variables			Model 1	Model 2
	OR [95% CI]	AIC	AOR^§^ [95% CI]	AOR^$^ [95% CI]
Gender (ref. = male)	0.79 [0.37, 1.68]	549.56		
Age	1.08 [1.05, 1.11]^*∗∗∗*^	509.27	1.15 [1.08, 1.24]^*∗∗∗*^	1.15 [1.07, 1.24]^*∗∗∗*^
Duration of exposure	1.28 [1.19, 1.37]^*∗∗∗*^	489.37	1.96 [1.20, 3.23]^*∗∗*^	2.01 [1.19, 3.39]^*∗∗*^
Age *∗* duration of exposure	1.00 [1.00, 1.01]^*∗∗∗*^	475.38	0.99 [0.98, 0.99]^*∗*^	0.99 [0.98, 0.99]^*∗*^
Ear plug (ref. = number)	0.47 [0.28, 0.77]^*∗∗*^	420.49	0.46 [0.26, 0.80]^*∗∗*^	0.40 [0.22, 0.71]^*∗∗*^

			AIC = 343.19	AIC = 328.56

^*∗*^ *p* < 0.05; ^*∗∗*^ *p* < 0.01; ^*∗∗∗*^ *p* < 0.001.

^§^Adjusted for type of quarry (place of work).

^$^Adjusted for type of quarry (place of work) and family history.

VIF = age (1.244); duration of exposure (1.250); ear plugs (1.047).
